# Comparison of a Deep Learning-Based Pose Estimation System to Marker-Based and Kinect Systems in Exergaming for Balance Training

**DOI:** 10.3390/s20236940

**Published:** 2020-12-04

**Authors:** Elise Klæbo Vonstad, Xiaomeng Su, Beatrix Vereijken, Kerstin Bach, Jan Harald Nilsen

**Affiliations:** 1 Department of Computer Science, Norwegian University of Science and Technology, 7034 Trondheim, Norway; xiaomeng.su@ntnu.no (X.S.); kerstin.bach@ntnu.no (K.B.); jan.h.nilsen@ntnu.no (J.H.N.); 2 Department of Neuromedicine and Movement Science, Norwegian University of Science and Technology, 7030 Trondheim, Norway; beatrix.vereijken@ntnu.no

**Keywords:** motion capture, image analysis, markerless motion capture, exergaming, segment lengths, kinect, deep learning, human movement

## Abstract

Using standard digital cameras in combination with deep learning (DL) for pose estimation is promising for the in-home and independent use of exercise games (exergames). We need to investigate to what extent such DL-based systems can provide satisfying accuracy on exergame relevant measures. Our study assesses temporal variation (i.e., variability) in body segment lengths, while using a Deep Learning image processing tool (DeepLabCut, DLC) on two-dimensional (2D) video. This variability is then compared with a gold-standard, marker-based three-dimensional Motion Capturing system (3DMoCap, Qualisys AB), and a 3D RGB-depth camera system (Kinect V2, Microsoft Inc). Simultaneous data were collected from all three systems, while participants (N = 12) played a custom balance training exergame. The pose estimation DLC-model is pre-trained on a large-scale dataset (ImageNet) and optimized with context-specific pose annotated images. Wilcoxon’s signed-rank test was performed in order to assess the statistical significance of the differences in variability between systems. The results showed that the DLC method performs comparably to the Kinect and, in some segments, even to the 3DMoCap gold standard system with regard to variability. These results are promising for making exergames more accessible and easier to use, thereby increasing their availability for in-home exercise.

## 1. Introduction

The proportion of older adults that are in need of guided physical exercise is expected to increase due to the coming demographic change. There is a need to develop technological tools that can aid and relieve clinicians in this effort [1,2]. In recent years, exercise gaming (exergaming) has emerged as a viable alternative, or addition, to traditional exercise. Exergames are designed to make the player move in a specific manner to train a specific function, such as stepping, sideways leaning, or moving their arms over their head. Typically, the person playing controls the game by moving their body: the game system captures their movements and uses this as input to control the game [3]. Exergames have also been shown to be more motivating and fun than traditional exercise [4,5], and they could potentially be used to provide quality, high-volume exercise guidance without having a physical therapist present to supervise [6,7].

Finding a motion capture tool that is suitable for this context is one of the areas in exergaming that is most challenging. If an older adult is going to use an exergame system, it needs to be user friendly, i.e., easy to understand and use efficiently, while providing input to the game that reliably represents the persons’ movements. The latter is a prerequisite for the exergame system being useful in a serious setting: accurate and reliable capture of a person’s movement is needed in order to ensure that the game is rewarding the player for correctly performed exercise movements and suggesting improvements to less-correctly performed movement patterns. This can facilitate good quality in performed movement patterns and increased motivation for exercise by providing appropriate rewards [8].

In this study, we investigate performance of a DL-based motion capture system by assessing the systems’ temporal variation (i.e., variability) in estimating body segment lengths as compared to the gold standard 3DMoCap system. In the remaining of this section, we describe the three relevant systems and the rationale of segment length variation as an exergame relevant measure.

In kinematic analysis in settings, such as in exergaming, the movement patterns of body segments are used as input to the game. Therefore, segment lengths are important to keep relatively constant to avoid erroneous representation of the player in the game. Segment lengths are also vital in scaling biomechanical models to the person being measured [9], and segment definitions affect the kinematic analysis of movement [10,11]. The most accurate tools, i.e., the gold standard for measuring human movement, are marker-based 3D motion capture systems (3DMoCap). These systems are expensive, in terms of cost, time, and knowledge required to use them, and they are, as such, infeasible to use in a person’s home.

One of the most popular tools for motion capture in exergaming is the Kinect (Microsoft Inc, Redmond WA, USA), a multiple-camera device while using RGB and depth (RGB-D) information in combination with machine learning-based (ML) analysis to detect human body parts and estimate three-dimensional (3D) joint positions of people, detected within the camera field of view [12]. In some contexts, Kinect cameras are useful, as they provide joint center position data directly with no need for additional processing of the depth or image data, as seen in, e.g., [13]. Kinect-based games vary in the gestures and movements that they elicit from the player. However, as there are many games that are designed for older adults, movements that challenge balance and posture control are common, as seen in [3]. Even though the joint center positions are not as accurately defined as in a 3DMoCap system [14], Kinect cameras have been shown to provide data that are sufficiently valid and reliable for some exergaming purposes [15,16]. However, we must be conscious of temporal variability in distal joints, such as wrists, elbows, knees, and ankles [17,18,19] when using Kinect-based systems for exergaming purposes. Additionally, even though Kinect cameras are more accessible than 3DMoCap systems, this still is an extra device that needs to be acquired and correctly set up before having access to exergames. This could be a potential barrier of use and it could be circumvented by utilizing standard digital cameras available in most homes today, such as smartphones, web cameras, and tablets.

Using standard digital video in motion capture has received increased attention in recent years, due to advances in DL-based image processing techniques. Frameworks, such as OpenPose [20], DeeperCut [21], and EfficientPose [22], provide kinematic information by extracting joint positions from video. DeepLabCut (DLC, [23]) is another interesting DL-based system that could potentially be used for pose estimation in humans. This has previously been used to reliably track points of interest on animals and insects in standard video and it could potentially be used to acquire human motion data during exergaming. One interesting feature of this framework is that it was shown to require a relatively low number of training samples in order to accurately predict joint center locations in unseen videos [23]. This is achieved by using transfer learning, which specializes the network to the specific context at hand. As noted in [24], using context-specific training data can optimize the pose estimation model, which might improve performance. This can potentially provide exergame users with a tool that performs with high accuracy in specialized settings, where the reliability of the exergame is vital in ensuring proper feedback and guidance. The framework is available through an open source, easy to use toolbox (github.com/deeplabcut/deeplabcut). Athough the above mentioned DL methods for pose estimation and joint tracking are evaluated for accuracy by comparing the estimated joint locations to the human pre-labelled training data, the tracking accuracy is rarely compared to a gold standard 3DMoCap system. This might be sufficient for their respective contexts, but, if we are to use such systems in settings such as exergaming in rehabilitation, where accuracy and stability in motion tracking are vital, we need to know how these systems perform when compared to the gold standard. Despite the potential of using standard digital video for motion tracking for exergaming, comparisons of a DL system, such as DLC to gold standard motion capture systems, has not yet been extensively investigated with regard to exergame relevant measures, such as variability of for example segment lengths. To our knowledge, the current study is the first to compare DLC to a 3DMoCap system and a Kinect camera system in terms of variability in segment lengths.

It is crucial for the further development and implementation of exergames to develop markerless motion capture systems that are easy to use and reliable to ensure proper feedback and guidance to the users during exergaming. In order to contribute to this goal, this study aims at comparing a transfer learning-based pose estimation model (DLC) to the Kinect system and a gold-standard 3DMoCap system.

The remainder of this paper is structured as follows: the methods and materials are found in Section 2. Section 3 details our results, and the discussion is found in Section 4.

## 2. Materials and Methods

### 2.1. Participants

We recruited healthy older adults from a local exercise group for seniors in Trondheim Norway. The inclusion criteria were age >65 years and the absence of physical or cognitive impairments or conditions that affected balance or gait ability. There were 12 participants in total (10F); the average age was 70.4 years (SD 11.4, range 54–92). The average height and weight were 172.3 (±11.4) cm and 70.4 (±12.1) kg, respectively. The exclusion criteria were physical or cognitive injuries/impairments that affected their balance or gait ability, and age <50 or age >80 years. The participants were given oral and written information regarding the study and gave their written consent, and the Norwegian Center for Research Data approved the study (reference number 736906). The participants attended one session each, and all completed the data collection without incident. The participants wore t-shorts and shorts of different types, colors, and fabrics, and some wore shorts and/or a sports bra.

### 2.2. Protocol

#### 2.2.1. The Exergame

**Figure 1:**
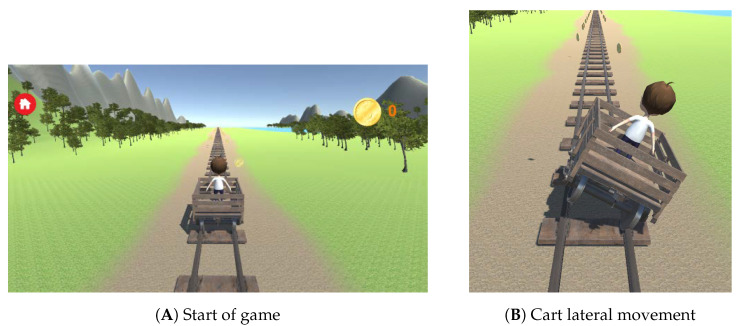
Screenshots from the game. (**A**) shows the start of the game, and (**B**) shows the cart leaning sideways with the movements of the player to hit coins along the track.

As medio-lateral weight-shifting movements are paramount in prevention of loss of balance function in older adults [25], and they are often used in Kinect-based games [26], a custom weight-shifting exergame was developed for the purpose of this study. The exergame was designed in order to elicit medio-lateral weight shifts from the participants: On the screen, an avatar in a rail cart was controlled by the participant, as seen in Figure 1A. If the participant leaned to one side the rail cart also tilted to the same side, allowing it to hit coins along the rail track as the cart moved down the track (Figure 1B).

#### 2.2.2. Equipment

**Figure 2:**
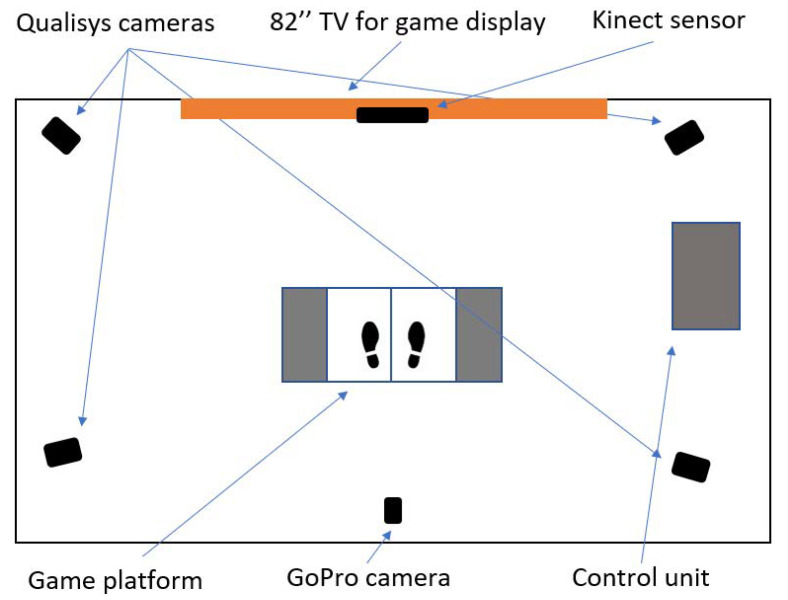
Experimental setup.

As input to control the game, a Kinect (v2, 30 Hz, Microsoft Inc., Redmond, WA, USA) camera system was used in order to track player movements. Participants’ movements were simultaneously measured while using 36 reflective markers placed according to the Plug-in-Gait Full-Body marker placement guide (PiG-FB, [27]) and a 3DMoCap system (90 Hz, Qualisys AB, Gothenburg, Sweden) consisting of four MX400 cameras, and a normal digital camera (30 Hz, 1400 × 720 px, GoPro Hero Black 3+, GoPro Inc., San Mateo, CA, USA) positioned 200 cm behind the center of the starting position of the participants. The participants’ height, weight, and age was also recorded. Figure 2 depicts a schematic of the experimental setup. The participants were standing on a 160 × 60 × 5 cm game platform while playing, where they had one force plate (Kistler Group, Winterthur, Switzerland) under each foot. Measurements from this equipment were not used in the current study.

### 2.3. Processing and Analysis

Processing was conducted while using Python (v 3.7), and statistical analyses were performed while using SPSS Statistics (v. 26, IBM Corp, Armonk, New York, NY, USA).

#### 2.3.1. Dataset

The current dataset consists of data from participants leaning from side to side to control the exergame. Each participant played six trials of the game, with each trial lasting for around five minutes. There is 3DMoCap data from 11 participants, DLC data from 12 participants, and Kinect data from 12 participants. In some participants (4, 8, 9, 10), the field of view of the GoPro camera resulted in ankles not being visible in the video. The ankle joints from these participants were excluded from analysis by the DLC system. 3DMoCap data from one participant (2) was corrupted and it was not included in the analysis.

#### 2.3.2. Preprocesssing of Kinect and 3DMoCap Data

**Figure 3:**
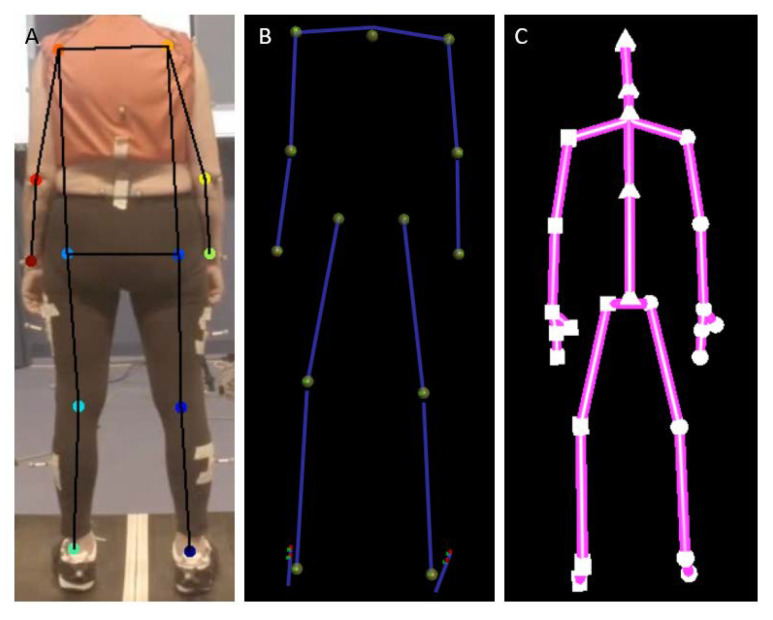
Joint centers as defined by the three motion capture systems (not to scale). (**A**) = DeepLabCut, (**B**) = 3DMoCap, (**C**) = Kinect.

The standard PiG-FB biomechanical model was used in order to extract joint center locations from 3DMoCap data. Joint center positions (distance to lab-coordinate system origin, mm) from shoulders, elbows, wrists, hips, knees, and ankles were extracted (Figure 3B). Normally, segment lengths (i.e., distance between joint center locations) are calibrated by the PiG-FB biomechanical model at the beginning of a trial and kept constant throughout the data capture. In this study, however, variability of segment lengths during the trial are of particular interest and will be used instead of the constant segment length calculated from the PiG-FB biomechanical model. For Kinect camera data, joint positions of shoulder, elbow, wrist, hip, knee, and ankle joints in the X and Y-axis (relative to the coordinate system origin within the camera itself) of the camera were extracted from the Kinect skeletal model (Figure 3C) using Kinect Studio (v. 2.0.14, Microsoft Inc) and the Kinect2Toolbox [28]. Because the data from Kinect were originally reported in meters, they was converted to millimeters to be comparable to DLC and 3DMoCap data.

#### 2.3.3. Preprocessing of DeepLabCut Data

The DLC framework is based on a feature detector method from one of the state-of-the-art human pose estimation frameworks, DeeperCut [21]. This employs a variant of ResNet that was pre-trained on the ImageNet [29] database. Semantic segmentation of images containing body parts is performed on all frames of the video data, and deconvolutional layers are used in order to up-sample the images after convolution and, thus, ensure sufficient spatial resolution for body part detection. Instead of the classification output layer of the CNN, score maps for the predictions of a body part in an image is produced. These spatial probability densities are then fine-tuned for each body part while using labelled images.

Digital video data were analyzed while using the DLC implementation software DeepLabCut (DLC, github.com/DeepLabCut/DeepLabCut). Joint center locations of shoulders, elbows, wrists, hips, knees, and ankles (Figure 3A) were manually applied to three images from two videos from each participant by an experienced human movement scientist, totaling 194 labelled images. This is in line with recommendations in [23]. These joint center locations in images were then used as training data for the neural network (ResNet101). The train/test split was set to 95/5. The DLC was trained for 220,000 iterations, with loss plateauing at 0.0012 at a p-cutoff of 0.01. After training, all of the videos were analyzed by the DLC, and the predicted joint center pixel locations were extracted. The DLC was then evaluated on the 5 % left out data to assess whether overfitting had occurred. To convert video pixel data to mm, the distance between shoulder joint centers were extracted from 3DMoCap data and then used as a reference to calculate pixel size. One pixel was found to be approx. 3.5 mm: the DLC pixel data was converted to mm by multiplying the pixel information with 3.5.

#### 2.3.4. Calculation of Segment Lengths and Variability

**Figure 4:**
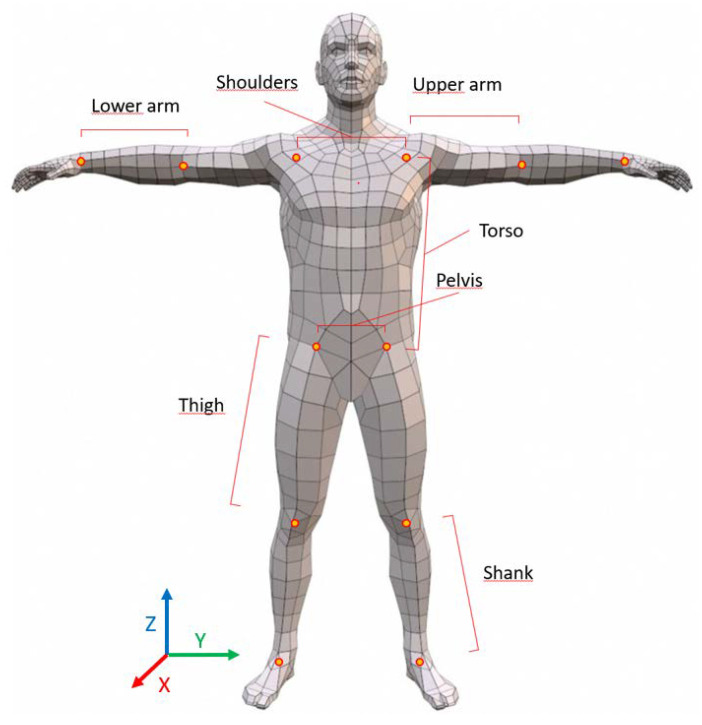
Joint locations, axes directions, and segment length definitions extracted from all three motion capture systems.

Segment lengths of shoulders, left and right upper arms, left and right lower arms, left and right torso sides, pelvis, left and right thighs, and left and right shanks were found by calculating the Euclidean distance between the joint centers for each joint set for each participant (Figure 4). Data points that were outside of mean ±3 standard deviations (SD) were considered to be outliers and removed from the dataset.

#### 2.3.5. Statistical Analysis

The 3DMoCap, Kinect, and DLC segment length variability were assessed by analyzing the Euclidean distance between joints in each time frame for each of the camera systems. To represent variability in these distances, standard deviation and coefficient of variation (coeffVar; dispersion of data around the mean) was employed. The Shapiro–Wilks test for normality gave a *p* < 0.05 for all segment lengths, which revealed that the data were not normally distributed. Therefore, the non-parametric Friedman test was conducted to assess statistical differences in segment length variability between the three systems. Subsequently, a post hoc analysis was conducted on the statistically significant differences from the Friedman test in order to extract which between-system differences were statistically significant. The post hoc analysis was conducted by using Wilcoxon’s signed-rank test with a Bonferroni correction, resulting in α = 0.017.

## 3. Results

**Figure 5:**
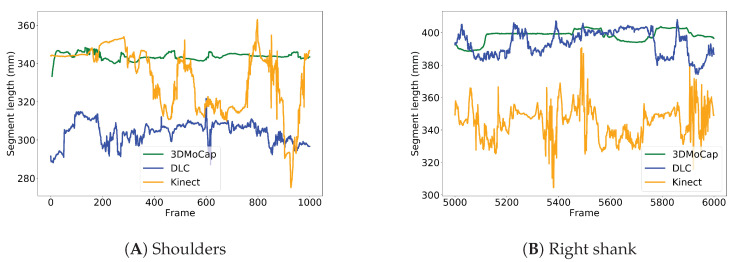
Comparison of variations in temporal segment lengths of the shoulder (**A**) and right shank (**B**). Image recording frequency 30 Hz.

**Figure 6:**
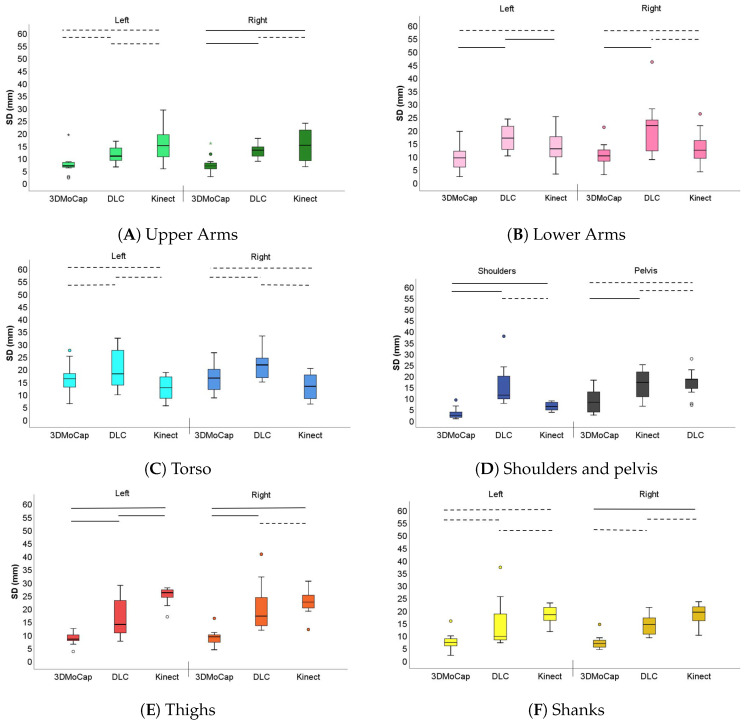
Box plots of variation of standard deviations of upper arms (**A**), lower arms (**B**), torso (**C**), shoulders and pelvis (**D**), thighs (**E**), and shanks (**F**) for the left and right side of the body. 3DMoCap = 3D motion capture system, DLC = DeepLabCut. Dotted lines signify *p* > 0.017, solid lines *p* < 0.017 from Wilcoxons Signed Rank test.

Table 1 shows the results from the mean segment lengths from each motion capture system. Table 2 shows the mean SD of segment lengths from each motion capture system. The results from the Friedman analyses of statistically significant difference can be found in Table 3. In Figure 5, a comparison of the variability of the segment length over 1000 frames of the shoulder and shank segments can be found. Figure 6 shows the results from the post hoc-test, as well as the median and IQR values as box plots.

### 3.1. Mean Lengths

Representative examples shown in Figure 5 depict temporal segment length variabilities in the three different motion capture systems. These are examples of the variability seen in segment lengths of the shoulder (Panel 5a) and right shank (Panel 5b) during 1000 frames, which corresponds to 33.3 s. The mean length of the shoulder segment that is seen in Figure 5A is approx. 344 mm, as measured by the 3DMoCap system with a range of approx. 15 mm. The DLC has a shorter segment (304 mm) with slightly higher variability (range 35 mm) when compared to the 3DMoCap system. The Kinect system starts with about the same segment length as the 3DMoCap system (333 mm), but shows increasing variability throughout the trial with a range of 88 mm. Differences in the average segment lengths are most likely due to different definitions of where the joint centers are located, as seen in Figure 3. The right shank segment (Figure 5B) shows similar results: the 3DMoCap system and the DLC measure similarly in mean shank length, at 393 mm and 398 mm, respectively, but the DLC has a larger range of 33 mm as compared to 15 in the 3DMoCap system. The Kinect system has a lower shank length, averaging at about 345 mm, and the variability is higher throughout the trial, with a range of 86 mm.

The mean segment lengths calculated from all data for all participants vary between the three motion capture systems, as shown in Table 1. This reflects the different biomechanical models mentioned earlier, although some segments are defined similarly and have similar lengths. Furthermore, the pelvis lengths show that, even though the Kinect system typically defines the hip joints more superior, or towards the head, as compared to the 3DMoCap system [14], the size of the segment is similar between the two systems. An example of this is the shoulder segment, where the difference between the Kinect and the 3DMoCap system is <2.5 mm, and approximately 20 mm between the DLC and the 3DMoCap. The Kinect system also seems to underestimate lower body segment lengths compared to 3DMoCap, while the DLC shows similar segment lengths here.

### 3.2. Segment Length Variability

Table 2 shows the variability of all systems for all segment lengths. This shows that the overall highest mean SD was 25.5 mm (Kinect, left thigh) and the overall lowest mean SD was 2.8 mm (3DMoCap, pelvis). The average of all mean SDs was 9.4 mm (SD 3.6) in the 3DMoCap system, 16.1 (SD 4.5) in the DLC system, and 16.4 mm (SD 5.1) in the Kinect system. The table also shows that the coeffVar was generally low, further indicating low variability in all three systems.

#### 3.2.1. Upper and Lower Arm

Figure 6A,B, shows arm segment variability, where panel A shows the upper arm and panel B shows the lower arm.

In the left upper arm (Panel A), the difference in SD between DLC (median 10.6 mm, IQR 8.2 to 14.0), and Kinect (median 14.8 mm, 7.5 to 19.2) is not statistically significant. The Kinect and the DLC system both had a higher SD than the 3DMoCap system (median 6.7 mm, IQR 5.9 to 8.2), but this was not statistically significant. In the right upper arm, the only difference in mean SD that is not statistically significant is between DLC (median 13.0 mm, IQR 10.3 to 14.8) and Kinect (median 15.0 mm, IQR 7.5 to 21.8), and both show statistically significant higher SD as compared to the 3DMoCap (median 6.7 mm, IQR 4.9 to 8.5).

In the left lower arm (Panel B), Kinect (median 12.7 mm, IQR 8.5 to 18.2) variability was not statistically different from the 3DMoCap (median 9.3 mm, IQR 4.8 to 11.9). However, the DLC (median 16.8 mm, IQR 12.0 to 21.7) showed statistically significant higher variability than the 3DMoCap and the Kinect. In the right lower arm, the DLC (median 21.5 mm, IQR 11.1 to 24.0) had statistically significant higher variability than 3DMoCap (median 10.0 mm, IQR 8.0 to 12.3), but not to the Kinect (median 12.2 mm, IQR 8.3 to 16.3).

#### 3.2.2. Torso and Shoulders

Panel C shows the median SD difference between the systems in the torso (left and right side). Here, the difference in mean SD was non-significant between any of the systems on neither the left (3DMoCap median 16.0 mm (IQR 11.4 to 18.3), Kinect median 12.4 mm (IQR 7.6 to 17.1) DLC median 18.0 mm (IQR 13.4 to 28.2)) nor the right side (3DMoCap median 16.3 mm (IQR 9.6 to 20.4), Kinect median 13.0 mm (7.2 to 18.6) DLC median 21.5 mm (IQR 16.4 to 26.6)). In the shoulder segment (Panel D) the difference between DLC (median 17.5 mm, IQR 8.8 to 23.0) and Kinect (median mm 18.7, IQR 13.2 to 18.9) is also non-significant. The median SD for the 3DMoCap shoulder segment was 8.7 mm (IQR 3.8 to 14.6).

#### 3.2.3. Pelvis

The pelvis segment SD (Panel D) difference was not statistically significant between the DLC (median 11.1 mm (IQR 9.4 to 21.0)) and Kinect (median 6.0 mm (IQR 4.2 to 8.0). The difference between the Kinect and 3DMoCap (median 2.1 mm, IQR 1.1 to 3.8) was not statistically significant.

#### 3.2.4. Thigh and Shanks

In panel E, the results for mean SD difference between the systems in the thigh segments are presented. These show that differences between systems are statistically significant on both the left and right thighs, except for DLC as compared to Kinect on the right side. The median SD of the thigh segment in 3DMoCap was 8.1 mm (IQR 7.6 to 9.9) and 9.1 mm (IQR 6.4 to 9.9) on the left and right side, respectively, while the median SD of DLC was 13.7 mm (IQR 10.5 to 23.1) and 17.0 mm (IQR 12.5 to 28.4), for the left and right side, respectively. The thigh median SD was the highest in the Kinect, with 25.9 mm (IQR 23.7 to 27.1) and 22.3 mm (IQR 18.9 to 15.8) on the left and right side, respectively.

On the left side, there are non-significant differences between 3DMoCap (median 7.0 mm, IQR 6.0 to 9.7) and DLC (median 9.5 mm, IQR 7.0 to 25.4), between DLC and Kinect (median 18.1 mm, IQR 15.1 to 21.9) and between 3DMoCap and Kinect, as seen in the results of shank segment variability (Panel F). On the right side, the difference between DLC (median 14.2 mm, IQR 9.5 to 19.1) and Kinect (median 19.1 mm, IQR 14.3 to 21.3) is not statistically significant, but the Kinect has a significantly higher mean SD than 3DMoCap (median 6.6 mm, IQR 5.0 to 8.9). The difference between DLC and 3DMoCap is not statistically significant.

## 4. Discussion

In this paper, we compared the DLC image analysis system to a Kinect system and a gold standard 3D motion capture system by studying the variability in segment lengths. Overall, the DLC method and Kinect system showed slightly higher variability than the 3DMoCap system, but this was not statistically significant for all of the comparisons. The highest mean SD found (25.5 in left thigh from Kinect, Table 2) shows that the systems generally perform with acceptable variability, even in the worst performing segment.

Our analyses show that the DLC variability difference is not statistically significant from the gold standard 3DMoCap system in several segments, namely left upper arm, left and right torso, and left and right shank. The Kinect variability is not statistically significantly different from the gold standard 3DMoCap system in the left upper arm, left and right lower arm, left and right torso, pelvis, and left shank. The difference in variability between the DLC and the Kinect is not statistically significant in the left and right upper arms, the left lower arm, left and right torso, shoulders, pelvis, right thigh, and left and right shanks.

### 4.1. Implications

The low variability of the DLC in predicting shank joint centers is promising for applying it in balance training settings while using weight-shifting movements, where stability in foot tracking is essential. This also applies to stepping exercises, which also constitute an important part of recommended exercises for older adults [30].

In the upper arms, the DLC and the Kinect systems both show slightly higher variability than the 3DMoCap system in the right arm. The Kinect showed highest variability, but this difference was not statistically significant when compared to the 3DMoCap. Reaching and leaning are often used in exercise for postural control and balance in older adults [31], and these results show that it might be feasible to use DLC in exergames that aim at eliciting such movements from players. The results from the shoulder segment also support this; even though the variability was highest in the DLC system, it was comparable to the Kinect.

Even though the hip joint centers often are difficult to track for marker-less models [32], our results show that the pelvis is tracked with a stability that is comparable to the 3DMoCap by both the Kinect and the DLC systems. This is also the case in the torso segments. They are able to reliably track the pelvis and torso segments is important in many exergames, as these often provide the base segments for assessing balance movements [33].

The thigh segment variability differences were statistically significant between all systems in both the left and right segment, except for the DLC as compared to Kinect in the right thigh. Here, the Kinect showed the highest median variability of all segments, while the DLC showed high IQR. The use of DLC in balance training for older adults is still feasible, as the variability is only slightly higher than in the 3DMoCap system. However, this needs to be taken into consideration by developers and clinicians who aim at introducing DL-based motion tracking into exergames for balance training, as there is some uncertainty of stability in the lengths of these segments.

### 4.2. Related Work

Our results are in line with previous studies on the validity of the Kinect system [14,15,17,18,34,35]. The Kinect generally performs with some difference to the gold standard 3DMoCap system, but within acceptable ranges in the contexts studied. We can compare our results to these studies, as pose estimation from (monocular) image data using other DL-based methods is an adjacent field of research. There are several methods utilizing ResNets in order to predict joint positions, as shown in Chen et al. [36]. These typically achieve Mean Per Joint Position Error (MPJPE, mm) of 48–108, which is comparable to our results of mean SD. Other DL methods achieve varying MPJPE [36], which ranges from 130 to 40, where the latter was achieved by Sun et al. [37] while using a volumetric representation of 3D pose by heat maps and joint regression. Furthermore, Arnab et al. [38] and Mehta et al. [39] developed models that were able to reach MPJPE of 54.3 mm and 63.6 mm, respectively, when testing on the Human3.6M dataset [40]. Despite the importance of knowing the temporal variability that a human pose estimation system has, there is limited research on the variability of segment lengths. It is reported in e.g., [18], where thigh, shank, upper, and lower arm variability was found to be higher in the Kinect when compared to the 3DMoCap system. Moreover, a similar study to ours was conducted by Nakano et al [41], where the results of using a variant of the OpenPose model showed a mean absolute error (MAE) of joint positions ranging from <5 mm to >40 mm when compared to 3DMoCap. Note that this study used five synchronized cameras for 3D OpenPose joint tracking and did not directly report variability. To our knowledge, this study is the first to evaluate the DLC system on variability in human pose estimation as compared to Kinect and 3DMoCap.

### 4.3. Further Considerations and Future Directions

Being aware of the potential positive and negative sides of different motion capture systems is vital when assessing whether a system is suitable for use in a given situation. Different measurement techniques can inherently influence data quality. Even though 3DMoCap is considered to be the gold standard for accuracy, it is prone to marker placement errors, soft issue artifacts, and marker occlusion [42,43]. Kinect based systems, or specifically the Kinect joint location algorithm, can have poor joint tracking in situations where a body part is not visible to the camera, unusual poses, or interaction with objects [18]. Previous research has extensively documented these weaknesses in both 3DMoCap and Kinect systems. However, because the DL-based systems in motion capture settings are still in their infancy, they have not yet undergone the scrutiny that widespread use gives. Therefore, the possible limitations and sources of error in DL methods, such as DLC, can be found in the technical details of how the method works. For instance, some CNN-based methods, such as DLC, are pre-trained on an enormous dataset (ImageNet, [29]) and then trained on data from specific contexts (such as in the current study) in the last stage of training. This means that the DLC might already be biased before it is trained on our context-specific image data, which could impact our results in unknown ways. Generally, DL models require such large datasets as ImageNet (>14.2 million images) in order to have sufficient training data for a given classification/estimation problem. However, the DLC system avoids this issue and it only requires around 200 images to predict joint centers in context-specific situations. This is possible due to pre-training, i.e., transfer learning, and the use of spatial probability densities for locating body parts in images (see [21,23] for details). This requirement of around 200 images is much more feasible to achieve for context-specific motion tracking, and it can make DL-based analysis within reach for users who do not have access to the normally required large-scale datasets for their applications.

Because DL models are restricted to learning from the training data that they see, the training data will significantly influence the outcome of the predictions that they make. In practice, this implies that, for a DLC system to be usable for any type of person, we need a dataset that represents all types of person in order to avoid errors that result from a non-representative dataset. This can be both a practical and an ethical issue in these systems, while being avoided in systems that (semi-)directly measure the points of interest, such as Kinect and 3DMoCap systems, or by using pre-trained networks. It is vital to build the knowledge required to introduce new technology into settings where users are particularly vulnerable, such as patient settings, where efficient time usage is critical for progress in regaining physical function. Errors or unintended bias in such systems can have consequences that are detrimental to a persons’ health or quality of life, which is why it is essential to highlight and discuss these issues in the context of motion capture for the rehabilitation or prevention of loss of physical function. Even though these issues are important to keep in mind when using DL systems, there is great potential in using such methods for motion capture in settings that require ease of use and where other motion capture systems are not feasible to use. An interesting future direction would be benchmarking DLC on large, variable datasets, and research is underway in order to investigate the performance of DLC in 3D settings with a larger variety in movement directions and types.

### 4.4. Limitations

There are some limitations to this study that are important to be aware of. There were only 12 participants, which might limit the generalizability of these results to the elderly population. Furthermore, the movements performed while playing the balancing exergame were mostly limited to the frontal plane of the participants. Other more complex movements might make the prediction of segment lengths more challenging. This is an important area for future research. The Kinect camera and the digital video camera were set up in their optimal configuration for capturing motion data from participants: the Kinect camera was in the anterior view of the frontal plane of participants and the GoPro in the posterior view of the frontal plane. However, the 3DMoCap system only consisted of four cameras, which led to missing information in some of the marker trajectories because of the occlusion of markers in one or more cameras, possibly contributing to the 3DMoCap system not achieving the performance possible in such systems.

### 4.5. Conclusions

Overall, these results are encouraging. The aim of introducing novel technological solutions in exergaming is to improve the cost efficiency and ease of use, thereby making exergaming more accessible for older adults or patients in the home or at a rehabilitation clinic. This is dependent on technological solutions that provide sufficient information regarding the person’s movement while exergaming. Using readily available digital cameras to track movement during exergaming could provide such a solution, thereby making it possible to use exergames without needing technical assistance. The results of the current study are the first to show that a DL-based motion capture system using transfer learning can achieve measurement stability in segment lengths that were comparable to a popular motion capture camera in exergaming for balance training, and, in some segments, even comparable to the gold-standard in motion capture.

Although 3DMoCap provides the best possible accuracy, in the trade-off between ease of use and accessibility versus accuracy, the former is given priority because of the requirements of the home/older adult context. In other situations, the difference between a DLC and a 3DMoCap might be considered too large, e.g., in a clinical gait analysis setting. The segment length variability that was found in this study would impact joint angle measurements, which results in the clinical assessment of the gait function of the patient potentially being altered. Such contexts will continue to require high accuracy in order to limit the risk of incorrect data, and marker-based 3DMoCap systems are therefore still the best option here—for the time being. Continuing research for improving marker-less systems is an important direction to take because of the potential benefits in resource use, usability, and flexibility. The results of the current study warrant further investigation into using DLC or similar systems in more complex movement patterns and other camera positions, and also implementing it in real-time in exergame settings.

## Figures and Tables

**Table 1:** Mean segment lengths (mm (1SD)). L = left, R = Right. *N* = data from number of participants. 3DMoCap = 3D motion capture system, DLC = DeepLabCut.

Segment	Side		3DMoCap		DLC		Kinect
		***N***		***N***		***N***	
Shoulders		11	328.8 (23.5)	12	308.8 (25.5)	12	330.9 (20.2)
Upper arm	L	11	269.5 (19.1)	12	351.0 (20.5)	12	269.8 (15.9)
	R	11	279.5 (22.6)	12	357.8 (23.2)	12	267.1 (13.2)
Lower arm	L	11	228.5 (20.4)	12	228.7 (16.6)	12	235.8 (13.3)
	R	11	225.1 (12.8)	12	231.9 (16.1)	12	235.3 (14.8)
Torso	L	11	444.7 (27.8)	12	568.5 (33.7)	12	503.8 (27.4)
	R	11	439.9 (25.9)	12	566.1 (38.3)	12	497.9 (27.6)
Pelvis		11	148.6 (5.6)	12	280.6 (28.5)	12	154.8 (9.5)
Thigh	L	11	409.0 (33.2)	12	405.9 (21.9)	12	373.8 (26.1)
	R	11	410.6 (33.0)	12	411.9 (27.1)	12	372.5 (29.4)
Shank	L	11	404.9 (23.4)	8	415.2 (34.0)	12	378.8 (29.0)
	R	11	402.8 (22.4)	8	414.4 (33.5)	12	374.3 (27.3)

**Table 2:** Mean standard deviation (mm, (coefficient of variation)) of segment lengths. L = left, R = Right. *N* = data from number of participants. 3DMoCap = 3D motion capture system, DLC = DeepLabCut. Light green = lowest mean SD within system; light red = highest SD within system. Bright green = overall lowest mean SD; bright red = overall highest mean SD.

Segment	Side		3DMoCap		DLC		Kinect
		***N***		***N***		***N***	
Shoulders		11	9.1 (0.02)	12	16.6 (0.04)	12	17.3 (0.05)
Upper arm	L	11	7.4 (0.03)	12	11.7 (0.04)	12	15.1 (0.05)
	R	11	7.3 (0.02)	12	13.0 (0.04)	12	15.2 (0.06)
Lower arm	L	11	9.6 (0.04)	12	14.4 (0.08)	12	13.7 (0.06)
	R	11	10.2 (0.04)	12	20.4 (0.08)	12	13.3 (0.05)
Torso	L	11	15.9 (0.03)	12	22.5 (0.04)	12	12.8 (0.02)
	R	11	15.7 (0.09)	12	22.5 (0.03)	12	13.1 (0.02)
Pelvis		11	2.8 (0.01)	12	7.3 (0.04)	12	6.1 (0.03)
Thigh	L	11	8.3 (0.02)	12	16.4 (0.03)	12	25.5 (0.06)
	R	11	8.7 (0.02)	12	20.5 (0.04)	12	23.1 (0.06)
Shank	L	11	8.6 (0.02)	8	14.5 (0.02)	12	21.1 (0.05)
	R	11	8.6 (0.02)	8	13.6 (0.02)	12	20.5 (0.05)

**Table 3:** Chi-square ($X^{2}$), *p*-value, and mean ranks from the Friedman test of statistical difference between mean segment length standard deviation. Df = degrees of freedom. L = left, R = Right. 3DMoCap = 3D motion capture system, DLC = DeepLabCut.

Segment	Side			Mean Rank		
		**$X^{2}$(df)**	***p***	**3DMoCap**	**DLC**	**Kinect**
Upper arm	L	3.8 (2)	0.148	1.55	2.09	2.36
	R	8.7 (2)	0.023	1.27	2.36	2.36
Lower arm	L	11.6 (2)	0.003	1.27	2.73	2.0
	R	7.81 (2)	0.020	1.45	2.64	1.91
Shoulders		11.1 (2)	0.004	1.18	2.45	2.36
Torso	L	5.6 (2)	0.060	1.91	2.55	1.55
	R	5.5 (2)	0.103	2.00	2.45	1.55
Pelvis		20.2 (2)	0.000	1.09	3.00	1.91
Thigh	L	16.5 (2)	0.000	1.18	1.91	2.91
	R	16.9 (2)	0.000	1.0	2.36	2.64
Shank	L	4.6 (2)	0.102	1.43	2.00	2.57
	R	8.9 (2)	0.012	1.14	2.14	2.71

## Abbreviations

The following abbreviations are used in this manuscript:

DL	Deep Learning
ML	Machine Learning
RBG-D	Red Blue Green - Depth
3DMoCap	3D Motion Capture
IMU	Inertial Measurement Unit
ResNet	Residual Neural Network
DLC	DeepLabCut
CNN	Convolutional Neural Network
SD	Standard Deviation
CoeffVar	Coefficient of Variation
